# Evaluation of dietary arginine supplementation to increase placental nutrient transporters in aged mares

**DOI:** 10.1093/tas/txad058

**Published:** 2023-07-05

**Authors:** Rafael E Martinez, Jessica L Leatherwood, Amanda N Bradbery, Brittany L Paris, Carolyn J Hammer, Dale Kelley, Fuller W Bazer, Guoyao Wu

**Affiliations:** Department of Animal Science, Texas A&M University and Texas A&M AgriLife Research, College Station, TX 77843, USA; School of Agricultural Sciences, Sam Houston State University, Huntsville, TX 77340, USA; Department of Animal Science, Texas A&M University and Texas A&M AgriLife Research, College Station, TX 77843, USA; Department of Large Animal Clinical Sciences, Texas A&M University, College Station, TX 77843, USA; Department of Animal and Range Sciences, Montana State University, Bozeman, MT 59717, USA; Department of Animal Science, Texas A&M University and Texas A&M AgriLife Research, College Station, TX 77843, USA; Department of Large Animal Clinical Sciences, Texas A&M University, College Station, TX 77843, USA; Department of Animal Sciences, North Dakota State University, Fargo, ND 58102, USA; Department of Veterinary Clinical Sciences, Oklahoma State University, Stillwater, OK 74078, USA; Department of Animal Science, Texas A&M University and Texas A&M AgriLife Research, College Station, TX 77843, USA; Department of Animal Science, Texas A&M University and Texas A&M AgriLife Research, College Station, TX 77843, USA

**Keywords:** aged, arginine, mare, placenta

## Abstract

Nine pregnant mares (18.2 ± 0.7 yr; 493.82 ± 12.74 kg body weight [**BW**]) were used to test the hypothesis that dietary supplementation of l-arginine would enhance placental vascularity and nutrient transport throughout gestation in aged mares. Mares were balanced by age, BW, and stallion pairing, and assigned randomly to dietary treatments of either supplemental l-arginine (50 mg/kg BW; *n* = 7) or l-alanine (100 mg/kg BW; *n* = 6; isonitrogenous control). Mares were individually fed concentrate top-dressed with the respective amino acid treatment plus ad libitum access to Coastal Bermudagrass hay. Treatments began on day 14 of gestation and were terminated at parturition. Mare BW, body condition score (**BCS**), and rump fat were determined, and body fat percentage was calculated every 28 d and concentrate adjusted accordingly. Doppler blood flow measurements including resistance index (**RI**) and pulsatility index for uterine artery ipsilateral to the pregnant uterine horn were obtained beginning on day 21 and continued every 7 d until day 154 of gestation, and prior to parturition. Parturition was attended with foaling variables and placental measures recorded. Placental tissue from the pregnant horn was analyzed histologically to assess cell-specific localization of vascular endothelial growth factor (**VEGF**) and cationic amino acid transporter 1 (SLC7A1) proteins. Semiquantitative analyses were performed using 10 nonoverlapping images per sample fixed in a 10× field (Fiji ImageJ v1.2). Mare performance data were analyzed using PROC MIXED in SAS and foaling and placental data were analyzed using PROC GLM. Gestation length at parturition was not influenced (*P* > 0.05) by supplemental arginine. Compared with arginine-supplemented mares, control mares had a thicker rump fat layer (*P* < 0.01) and greater percent body fat (*P* = 0.03), and BCS (*P* < 0.01) at parturition. Arginine-supplemented mares had a lower RI than control mares prior to parturition (*P* < 0.01). Body length, height, and BW of foals at birth, as well as placental weight and volume, and immunohistochemical staining for VEGF and SLC7A1 at parturition, were not affected (*P* > 0.05) by maternal arginine supplementation. These results indicate that dietary arginine supplementation (50 mg/kg BW) is safe for gestating mares. A larger number of mares is required to extend knowledge of effects of supplemental arginine on embryonic/fetal survival and growth in mares.

## Introduction

Aged broodmares valued for reproduction generally possess impressive black-type pedigree and impeccable genetics. However, advancing age negatively impacts the reproductive capacity of these valuable mares, as their uterine endometrium undergoes degeneration that does not favor implantation and placental development (Bracher et al., 1996; Wilsher and Allen, 2003; Fowden et al., 2013; Robles et al., 2022). The conceptus requires that amino acids for protein accretion, metabolic processes, and other biosynthetic pathways be delivered across the uteroplacental interface through maternal and fetal blood exchange (Silver et al., 1994; Wu et al., 2009, 2018). Advanced age and the resulting degenerative changes in the uterus may impact this nutrient exchange.

The dietary supplementation of l-arginine enhances placental development and reproductive performance in other species (Wu et al., 2013). Unfortunately, there is limited research regarding requirements of amino acids for the horse, particularly of requirements for l-arginine. Kelley et al. (2013) revealed dietary supplementation of mares with 100 g of l-arginine increased ovarian blood flow and the size of dominant follicles 10 d after the preceding ovulation and reduced the accumulation of uterine fluid postbreeding. Köhne et al. (2018) reported that arginine supplementation at 0.0125% of body weight (**BW**) supports embryonic growth as fetal size from days 25–45 after ovulation was greater in both younger and older mares (young: 12.4 ± 0.8 mm and old: 14.3 ± 1.4 mm) when compared to nonsupplemented control mares (young: 10.6 ± 0.6 mm and old: 11.4 ± 0.8 mm). Furthermore, primiparous mares supplemented with 100 g/d of l-arginine during late gestation produced foals with heavier birthweights than primiparous mares that were not supplemented with l-arginine (Chavatte-Palmer et al., 2018).

To date, placental expression of vascular endothelium growth factor (**VEGF**) has not been detected in multiparous mares supplemented with l-arginine compared to primiparous mares (Robles et al., 2018), and there are no reports of expression of VEGF in placentae from aged mares. Allen et al. (2007) reported that VEGF facilitates development of the extensive fetal and maternal capillary networks that are prominent features within the microcotyledons of the diffuse, epitheliochorial equine placenta. Those results demonstrate the importance of VEGF in facilitating development of maternal and fetal vascular networks for the interchange of gases, nutrients, and waste products throughout gestation. Furthermore, VEGF has been reported to increase the transport of arginine via modulation of **SLC7A1** (transporter of lysine and arginine) expression in endothelial cells (Shashar et al., 2017).

Most mammals (e.g., humans, pigs, and rats) can convert glutamine, glutamate, and proline into ornithine, citrulline, and arginine in their enterocytes (Wu and Morris, 1998; Wu et al., 2004). Impaired placental development in older broodmares along with their inability to endogenously synthesize citrulline and l-arginine from glutamine, glutamate and proline (Martinez et al., 2022), as well as the lack of our knowledge about their dietary requirements for arginine, indicates the scientific importance of this information for equine researchers, nutritionists, and producers. l-Arginine is of particular interest due to its multiple biological responsibilities that are critical for adequate placental growth and fetal development throughout pregnancy (Wu et al., 2021). Interestingly, there is no de novo synthesis of l-arginine by enterocytes and there is no established requirement of dietary l-arginine for aged pregnant mares or any horse during their various stages of life (Martinez et al., 2022). Based on the foregoing literature review, there is no study concerning the role of maternal dietary arginine supplementation for aged mares on placental vascularity and the expression of the angiogenic factor VEGF or SLC7A1 for transport of arginine by endothelial cells of the placenta. Therefore, the present study tested the hypothesis that dietary supplementation of arginine (50 mg/kg BW/d) to aged mares throughout gestation would improve the utero-placental environment and indices of improved reproductive performance.

## Materials and Methods

All care, handling, and sampling of horses were reviewed and approved by the Institutional Animal Care and Use Committee at Texas A&M University (2018-0426).

### Horses and Management

Nine open and dry multiparous mares (mean ± SEM; 18.22 ± 0.68 yr; 5.8 ± 0.22 body condition score (**BCS**); 493.82 ± 12.74 kg BW) from an established herd were used in this study. Daily observation for signs of estrus was determined by teasing mares with a stallion and follicular development and ovulation were monitored daily using B-Mode (grayscale) ultrasonography with a rectal probe and rectal palpation of the ovaries. Following the first estrous cycle of the season, each mare was inseminated with fresh semen from one of two fertile stallions that belong to Texas A&M University. Semen was collected using a Missouri-Style artificial vagina and evaluated for concentration of sperm cells and total progressive motility by a single investigator. Each insemination dose consisted of a dose of 500 × 10^6^ fresh, progressively motile spermatozoa extended in INRA 96 (IMV Technologies, L’Aigle, France) in a total volume of 30 mL. Inseminations occurred 24 h following 1 mL deslorelin acetate injection (SucroMate, Dechra Veterinary Products, Louisville, KY), and inseminations continued every other day until ovulation. A Chison ECO (ECO 5, Xinwu District, Wuxi, Jiangsu, China), a 10–5-MHz broadband, transrectal R7-A transducer was utilized to scan ovaries of mares to monitor follicular development, ovulation, and pregnancy.

### Treatments and Housing

Dietary treatments began when pregnancy was confirmed on day 14 postovulation and ended on the day of parturition. Mares were assigned randomly to dietary treatments and stratified by age, BW, BCS, and stallion pairing. Mares (*n* = 5) received a dietary supplement of either 50 mg/kg BW of arginine (treatment; ARG) per day, or the same diet supplemented with 100 mg/kg BW of l-alanine (*n* = 4) to achieve isonitrogenous diets (control; CON). Arginine supplementation rate in the current study was based on amounts yielding positive effects without evidence of impairment to amino acid absorption, decreases in uterine fluid accumulation, or large fetuses (Kelley et al., 2014; Mesa et al., 2015; Köhne et al., 2018).

Throughout the initial two trimesters of gestation, mares were individually fed a commercial concentrate at 1.18% BW/d (as-fed basis) (Triumph Active 12% Pellet Horse Feed, Nutrena, Minneapolis, MN) split into two equal meals per day using individual feeding bags (Derby Originals Breathable Canvas Feed Bag with No-Spill Design, Royal International LLC, North Canton, OH). l-Arginine or l-alanine (99–100% pure, Ajipure, Ajinomoto AminoScience LLC, Raleigh, NC, USA) was top-dressed onto the concentrate and thoroughly mixed immediately prior to feeding once daily. Mares were allowed 50 min to consume the concentrate and respective dietary treatments. No refusals were recorded, ensuring that all mares individually consumed their respective meal and dietary treatment. Once mares entered their third trimester, their intake of concentrate increased to 1.25% BW. Mares were fed throughout the study according to 2007 National Research Council’s recommendations for feeding pregnant mares relative to stage of gestation (NRC, 2007). Grain intake for mares was adjusted every 28 d according to changes in BW.

All mares were group-housed in dry lots (58.7 × 79.2 m) and kept under natural light at the Texas A&M University Equine Center where they had ad libitum access to forage in the form of round bales of Coastal Bermudagrass (*Cynodon dactylon*) hay, water, and trace mineral salt. Composited grain and hay samples obtained from the same batch utilized throughout the entire study, were analyzed by a commercial laboratory (Equi-Analytical Laboratories, Ithaca, NY) for nutrient composition (Table 1), and in-house for amino acid composition by high-performance liquid chromatography as previously described (Wu et al., 1995; Table 2).

**Table 1.** Analysis of nutrient composition of the commercial concentrate and Coastal Bermudagrass hay offered to pregnant mares in this study

Nutrient^1^	Concentrate^2^	Coastal Bermudagrass hay^3^
DE, Mcal/kg	2.91	1.66
CP, %	15.90	9.30
Crude fat, %	7.40	0.90
NDF, %	33.30	69.00
ADF, %	19.30	42.50
Starch, %	23.80	3.60
Ca, %	1.32	0.49
P, %	0.83	0.14
K, %	1.16	0.97
Mg, %	0.39	0.12
Na, %	0.43	0.01
Cl, %	0.73	0.19
S, %	0.25	0.24
Co, mg/kg	1.77	2.32
Fe, mg/kg	268.00	598.00
Zn, mg/kg	208.00	38.00
Cu, mg/kg	45.00	13.00
Mn, mg/kg	163.00	44.00

ADF: acid detergent fiber; Ca: calcium; Cl: chlorine; Co: cobalt; CP: crude protein; Cu: copper; DE: digestible energy; Fe: iron; K: potassium; Mg: magnesium; Mn: manganese; Na: sodium; NDF: neutral detergent fiber; P: phosphorus; S: sulfur; Zn: zinc.

^1^Values presented on a 100% DM basis.

^2^Concentrate = basal grain diet fed to all horses at 1.18% up to 1.25% BW (as-fed basis) per day with advancing gestation (Triumph Active 12% Pellet Horse Feed, Nutrena).

^3^Coastal Bermudagrass (*Cynodon dactylon*) hay was offered ad libitum.

**Table 2.** Amino acid composition of the commercial concentrate and Coastal Bermudagrass hay offered to pregnant mares in the present study based on HPLC analyses

Amino acid^1^	Concentrate^2^	Coastal Bermudagrass hay^3^
Aspartate + asparagine	7.92	5.89
Glutamate + glutamine	18.21	5.68
Serine	5.11	2.27
Histidine	2.63	0.61
Glycine	4.73	2.97
Threonine	3.72	2.30
Arginine	5.50	2.33
Alanine	6.20	3.08
Tyrosine	3.85	2.28
Methionine	1.59	0.31
Valine	5.16	2.94
Phenylalanine	4.47	2.48
Isoleucine	3.79	2.25
Leucine	8.42	4.02
Lysine	8.42	7.82

^1^Values presented on a g/kg of diet (as-fed basis). Amino acids in the feedstuffs were determined after acid hydrolysis (Wu and Knabe, 1994; Wu and Meininger, 2008)

^2^Concentrate = basal grain diet fed to all horses at 1.18% up to 1.25% BW (as-fed basis) per day with advancing gestation (Triumph Active 12% Pellet Horse Feed, Nutrena).

^3^Coastal Bermudagrass (*Cynodon dactylon*) hay was offered ad libitum.

### Mare Measurements

Mare BW, BCS, and rump fat (**RF**) measurements were recorded every 28 d until parturition. Body weight was recorded using a calibrated platform scale (Bastrop Scale Inc., Bastrop, TX). One trained investigator determined BCS on a scale of 1 to 9 as described by Henneke et al. (1983) with 1 = poor and 9 = extremely fat. Rump fat was measured via ultrasonic images (ECO 5) on the left hip at a point 5 cm dorsal to the halfway point between the first coccygeal vertebrae and the ischium (Westervelt et al., 1976). Body fat predicted by RF thickness was calculated using the prediction equation developed by Westervelt et al. (1976) as follows: body fat (%) = 8.64 + 4.70 × rump fat thickness (RFT) (cm).

### Uterine and Fetal Ultrasonography

Color Doppler ultrasonography was performed transrectally to measure blood flow to the reproductive tract beginning on day 21 and then every 7 d until day 154 of gestation when microcotyledon development ceases (Samuel et al., 1974, 1975), and again prior to parturition. Transrectal examinations of blood flow to the reproductive tract of all mares took place between 0800 and 1000 h. Times at which Doppler measurements were obtained coincide with peak postprandial concentrations of arginine in plasma (Kelley et al., 2014).

Blood flow measurements were calculated as pulsatility index (**PI**) and resistance index (**RI**) in the uterine artery ipsilateral to the pregnant uterine horn; defined as the gravid uterine artery (**GUA**). The arteries were identified based on anatomical descriptions of Bollwein et al. (1998). Doppler blood flow measurements for uterine arteries were determined using an algorithm package of the Chison ECO (ECO 5) with a 10–5-MHz broadband, transrectal R7-A transducer.

### Foaling Variables

Nine mares were observed at parturition (ARG, *n* = 5 and CON, *n* = 4). Mares were monitored for signs of impending parturition and housed in individual stalls when foaling appeared to be imminent. Coastal Bermudagrass (*C. dactylon*) hay was offered ad libitum to all mares. Foalings (*n* = 9) were attended, and foaling variables were recorded including gestation length and time from release of fetal fluids via the vagina to birth (fetal expulsion) from birth to placental expulsion, from birth to standing, and from birth to nursing. Immediately following parturition, BWs of foals were taken prior to nursing. Additionally, colostrum refractometer (equine colostrum refractometer, Animal Reproduction Systems, Chino, CA) readings provided measurements of Brix percentage as an indirect measurement of IgG in colostrum prior to suckling by the foal. At 12 h after parturition, BW, body length, wither height, and hip height of foals were determined. None of the mares experienced dystocia or retained placenta.

### Collection and Fixation of the Placentae

At parturition, time of placental expulsion, placental weight, and placental volume were recorded. To measure placental volume, the entire placenta (allantochorion and amnion) was submerged in a graduated cylinder with known volume of ddH_2_O. After allowing for the ingress of water (≤5 min) into any air spaces, the volume of water displaced was measured (Wilsher and Allen, 2003). The total displacement was summed to calculate the volume of the entire placenta in milliliters. Placental samples from the same location in the pregnant uterine horn were snap frozen in liquid nitrogen and stored at −80 °C for protein analyses.

### Immunohistochemistry Sample Analysis

Placental samples (2 × 2 in) from the tip of the corresponding pregnant uterine horn were fixed in 4% paraformaldehyde and used for immunohistochemical analyses, as we described previously (Elmetwally et al., 2022). Sections (5 μm) of paraffin embedded placental samples were mounted to glass slides. Antigen retrieval was performed using boiling 0.01 M sodium citrate buffer (pH 6.0) for VEGF using a rabbit *VEGF* polyclonal antibody (Catalog No. 19003-1-AP; Thermo Fisher Scientific) at a 1:150 dilution, as well as a rabbit Anti-CAT1 antibody (Catalog No. ab37588; Abcam, Cambridge, UK) at a dilution of 1:150. Purified nonrelevant rabbit IgG was used as a negative control. Immunoreactive protein was visualized using the Vectastain ABC Kit (Catalog No. PK 6101 for rabbit IgG; Vector Laboratories, Burlingame, CA) following the manufacturer’s instructions and 3,3'-diaminobenzidine tetrahydrochloride (Catalog No. D5637; Sigma-Aldrich, St. Louis, MO) was used as the color substrate. Sections were prepared with a hematoxylin counterstain, and a coverslip was fixed using Permount mounting medium (SP15-500; Thermo Fisher Scientific). Digital images were captured using a Nikon DS-Ri1 camera equipped with NIS-Element AR 4.30.02 software. Semiquantitative analyses were performed using 10 nonoverlapping images per sample in a 10× field (Fiji ImageJ v1.2).

### Statistical Analysis

Data for all mares (ARG, *n* = 5 and CON, *n* = 4) were analyzed using PROC MIXED in SAS v9.4 (SAS Inst., Inc., Cary, NC). All data were analyzed as a randomized design with main effects being treatment, day, and treatment × day interaction. The model contained main effects of diet and day, the diet × day interaction, and a random effect of mare(diet). Where day 21 values differed by treatment, day 21 was included in the model as a covariate for PI and RI. Data for foaling performance and placental variables (ARG, *n* = 5 and CON, *n* = 4) were analyzed using the GLM procedure of SAS and the model contained the main effect of treatment. Significant LS mean differences were obtained by utilizing pairwise difference statement for treatment, and day.

Foal sex and sire were used in the statistical model, but were not significant; therefore, those variables were removed to conserve degrees of freedom. All data are presented as least squares means ± SEM. Main effects were considered significant when *P* ≤ 0.05 and were considered as a trend toward significance when *P* ≤ 0.10.

## Results

### Feed Intake of Mares

There were no treatment effects on concentrate intake throughout gestation (*P* = 0.52), as concentrate intake averaged 6.34 ± 0.06 kg/d for CON mares and 6.29 ± 0.06 kg/d for ARG supplemented mares. The intake of concentrate increased over time (*P* ≤ 0.05) as BW of mares increased with advancing gestation.

### Mare Performance During Gestation

**Figure 1.**
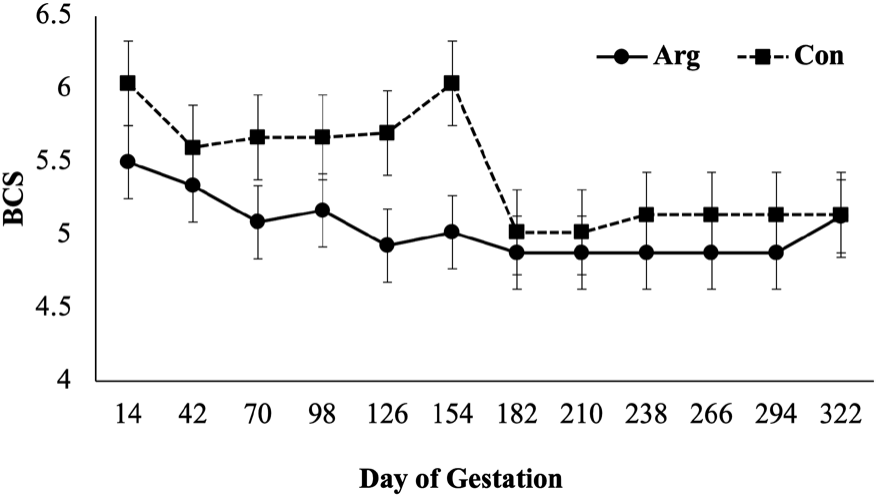
Body condition score of aged mares supplemented with either 50 mg/kg body weight (BW) of arginine (treatment; ARG, *n* = 5), or the same diet supplemented with 100 mg/kg BW of l-alanine to achieve isonitrogenous diets (control; CON, *n* = 4) throughout gestation. Main effects included diet (*P* = 0.34), day (*P* < 0.01), and diet × day (*P* = 0.02). * Within time point, refers to a dietary difference (*P* < 0.05).

**Figure 2.**
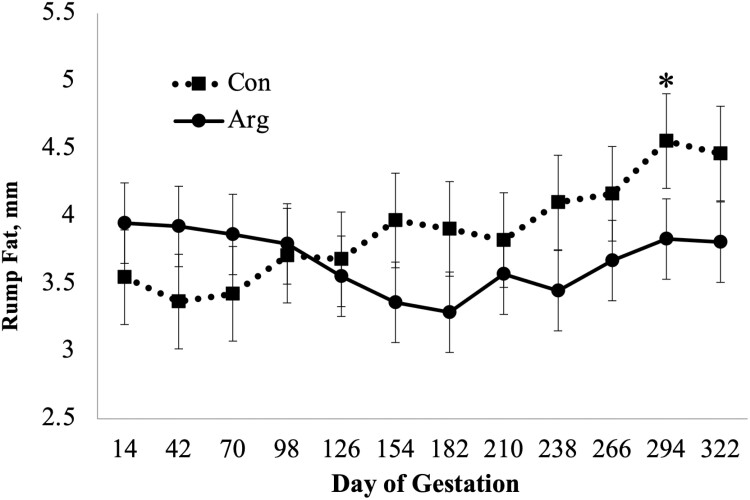
Rump fat thickness of aged mares supplemented with either 50 mg/kg body weight (BW) of arginine (treatment; ARG, *n* = 5), or the same diet supplemented with 100 mg/kg BW of l-alanine to achieve isonitrogenous diets (control; CON, *n* = 4) throughout gestation. Main effects included diet (*P* = 0.52), day (*P* = 0.34), and diet × day (*P* = 0.03). * Within time point, refers to a dietary difference (*P* < 0.05).

**Figure 3.**
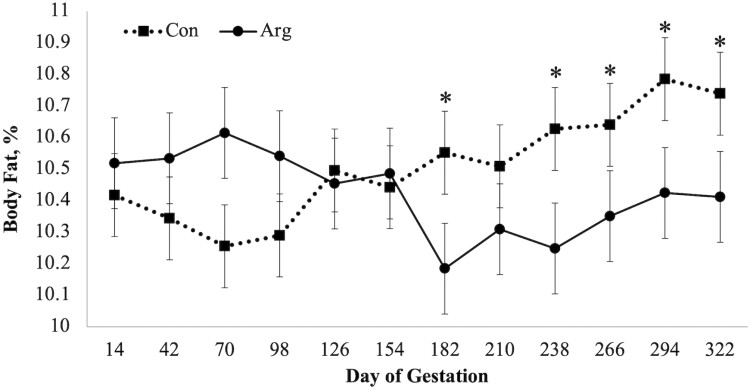
Estimated percent body fat of aged mares supplemented with either 50 mg/kg body weight (BW) of arginine (treatment; ARG, *n* = 5), or the same diet supplemented with 100 mg/kg BW of l-alanine to achieve isonitrogenous diets (control; CON, *n* = 4) throughout gestation. Main effects included diet (*P* = 0.13), day (*P* = 0.93), and diet × day (*P* = 0.03). * Within time point, refers to a dietary difference (*P* < 0.05).

**Figure 4.**
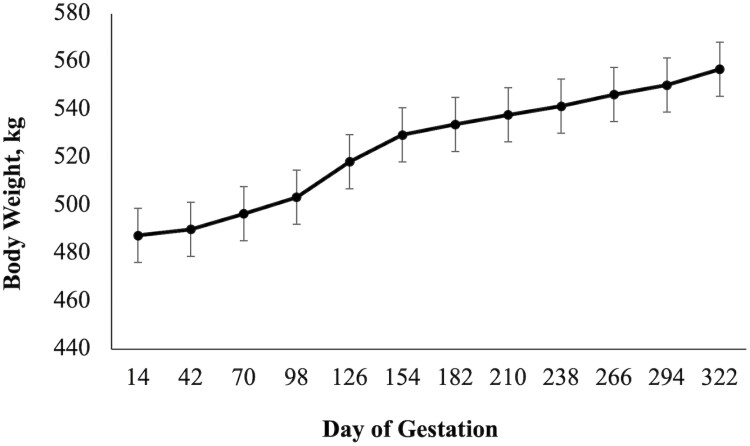
Body weight of aged mares from day 14 to parturition. Due to the lack of effect of a diet or diet × day interaction, dietary treatments were combined (*n* = 9). Main effects included diet (*P* = 0.70), day (*P* < 0.01), and diet × day (*P* = 0.36).

**Figure 5.**
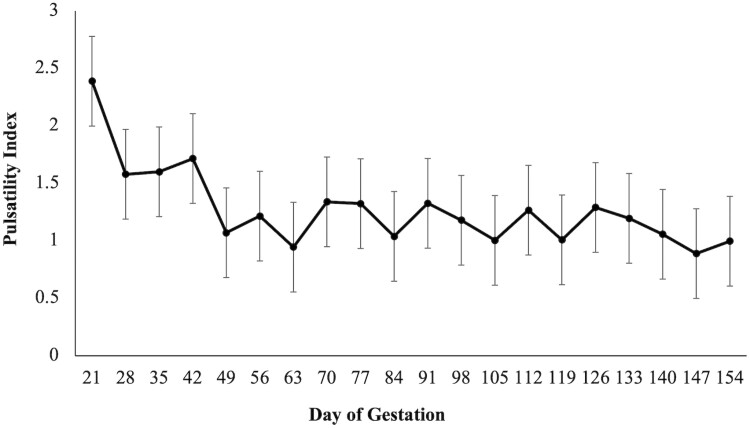
Pulsatility index of the gravid uterine artery in aged mares during the initial 154 d of gestation. Due to the lack of effect of a day, diet or diet × day interaction, dietary treatments were combined (*n* = 9). Main effects included diet (*P* = 0.26), day (*P* = 0.39), and diet × day (*P* = 0.44).

**Figure 6.**
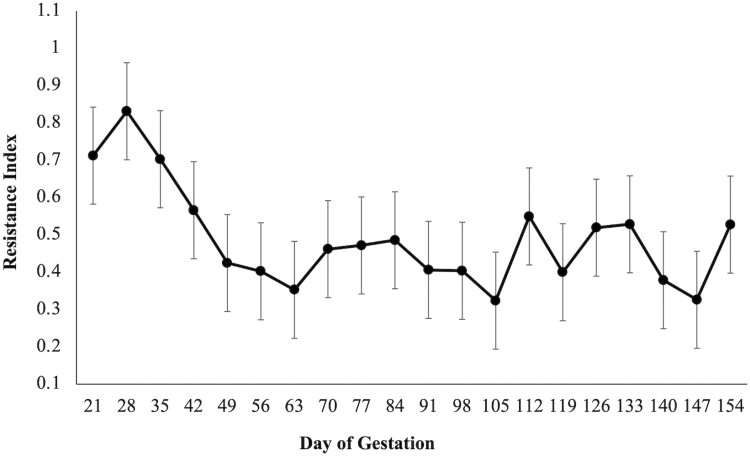
Resistance index of the gravid uterine artery in aged mares during the initial 154 d of gestation. Due to the lack of effect of a day, diet or diet × day interaction, dietary treatments were combined (*n* = 9). Main effects included diet (*P* = 0.28), day (*P* = 0.32), and diet × day (*P* = 0.77).

There was a diet × day interaction (*P* = 0.02; Figure 1) on BCS as CON mares had a higher BCS when compared to ARG mares on days 126 and 154. A diet × day (*P* = 0.03; Figure 2) was observed for RF, as CON mares had a higher RF when compared to mares in the ARG group, specifically noted on day 294. Similarly, a diet × day (*P* = 0.03, Figure 3) interaction was also observed for percent body fat, as ARG mares had a higher body fat percentage on day 70. As gestation progressed, the CON mares had a higher percentage of body fat on day 182 and on day 238 through parturition. Furthermore, the BW of all mares increased (*P* < 0.01) as they approached parturition regardless of dietary treatment (*P* = 0.70; Figure 4). Finally, there was no main effect of diet or day on PI (*P* = 0.26; Figure 5) or RI (*P* = 0.28; Figure 6) of the uterine artery ipsilateral to the pregnant uterine horn during the initial 154 d of gestation.

### Mare Performance to Parturition

A dietary affect was observed for BCS (*P* < 0.01; Table 3), as CON mares had a higher BCS throughout gestation when compared to ARG mares at parturition. Additionally, mares fed the arginine-supplemented diet had lower RI the day prior to parturition (*P* = 0.01; Table 3) for the uterine artery ipsilateral to the pregnant uterine horn when compared to CON mares. However, there was no dietary influence on rump fat thickness (*P* = 0.67), gestation length (*P* = 0.84), pre- (*P* = 0.79) and postpartum BW (*P* = 0.74), percentage of body fat (*P* = 0.70), or pulse index (*P* = 0.25) at parturition (Table 3).

**Table 3.** The effect of arginine^3^ supplementation to aged pregnant mares and their performance at pre- and postpartum

Foaling performance^1^	Diet		*P*-value
	CON^2^ (*n* = 4)	ARG^3^ (*n* = 5)	
Length of gestation, d	337.2 ± 5.7	338.8 ± 5.1	0.84
Prepartum BW, kg	554.7 ± 19.6	547.4 ± 17.8	0.79
Postpartum BW, kg	490.7 ± 19.8	499.1 ± 17.7	0.74
BCS^4^	5.9 ± 0.1^a^	5.3 ± 0.1^b^	<0.01
Rump fat, mm	4.2 ± 0.3	4.1 ± 0.3	0.67
Body fat, %^5^	10.6 ± 0.1	10.6 ± 0.1	0.70
Pulse index^6^	2.8 ± 0.3	2.2 ± 0.3	0.25
Resistance index^6^	1.2 ± 0.1^a^	0.9 ± 0.1^b^	0.01

BCS: body condition score.

^1^Values (mean ± SEM).

^2^Alanine supplementation (isonitrogenous control) at 100 mg/kg BW/d (*n* = 4).

^3^Arginine supplementation at 50 mg/kg BW/d (*n* = 5).

^4^Obtained using the 1–9 scale (Henneke et al., 1983).

^5^Percent body fat predicted by rump fat thickness.

^6^Blood flow measurements obtained as pulsatility index and resistance index of the uterine artery ipsilateral to pregnant uterine horn, defined as the gravid uterine artery, prior to parturition.

^a^,

^b^Differences (*P* ≤ 0.05) in dietary treatment.

### Foaling Variables

Allmares delivered normal, healthy foals without difficulty or placental retention. Foal BW at parturition and 12 h postpartum was not influenced (*P* = 0.92; Table 4) by maternal dietary supplementation of arginine. Foal size parameters (e.g., wither height, hip height, or body length) and brix percentage evaluation of colostrum were not influenced (*P* = 0.47; Table 4) by dietary treatment. Additionally, placental expulsion and foal standing times were not different (*P* = 0.24) between diets. Placental variables (Table 5) were not affected by the dietary supplementation of arginine throughout gestation (*P* = 0.23).

**Table 4.** The effect of arginine^3^ supplementation to aged pregnant mares and the performance of their foals at parturition and at 12 h postparturition

Foaling variables^1^	Diet		*P*-value
	CON^2^ (*n* = 4)	ARG^3^ (*n* = 5)	
Pre-suckle BW, kg	40.6 ± 3.4	40.6 ± 3	0.99
12 h BW, kg	42.9 ± 3.6	42.6 ± 3.2	0.92
Wither height, cm	94.8 ± 2.1	97 ± 2.1	0.47
Hip height, cm	94.5 ± 2.7	97.3 ± 2.7	0.52
Body length, cm	71.1 ± 3	70.1 ± 3	0.76
Colostrum^4^	23 ± 1.7	25.2 ± 1.5	0.35
Standing time, min	37 ± 3.7	32.4 ± 3.3	0.38
Placental expulsion, min	67 ± 13	44.2 ± 11.6	0.24

BW: body weight.

^1^Values (mean ± SEM).

^2^Alanine supplementation (isonitrogenous control) at 100 mg/kg BW/d (*n* = 4).

^3^Arginine supplementation at 50 mg/kg BW/d (*n* = 5).

^4^Brix Refractometer Scale (0–32%).

**Table 5.** Effect of arginine^3^ supplementation to aged pregnant mares on the placentae at term

Placental variables^1^	Diet		*P*-value
	CON^2^ (*n* = 4)	ARG^3^ (*n* = 5)	
Placental weight, kg	4.1 ± 0.3	4.4 ± 0.3	0.56
Placental volume, mL	4000 ± 342.1	4125 ± 306	0.40
Umbilical length, cm	62.6 ± 5.1	69.6 ± 4.6	0.34
VEGF^4^	7.6 ± 2	10.1 ± 1.7	0.39
SLC7A1^4^	1.4 ± 1.3	3.6 ± 1.1	0.23

SLC7A1: cationic amino acid transporter 1; VEGF: vascular endothelial growth factor.

^1^Values (mean ± SEM).

^2^Alanine supplementation (isonitrogenous control) at 100 mg/kg BW/d (*n* = 4).

^3^Arginine supplementation at 50 mg/kg BW/d (*n* = 5).

^4^Data obtained following semiquantitative analyses were performed using 10 nonoverlapping images per sample fixed in a 10× field (Fiji ImageJ v1.2) High affinity cationic amino acid transporter 1 of lysine and arginineand vascular endothelial growth factor.

## Discussion

Although the BCS were statistically different throughout different periods of gestation, body condition scores for mares in each treatment group were always about 5. Optimal BCS during gestation is 6 (Lawrence et al., 1992), but limited information is available for optimal BCS during pregnancy for mares of advanced age. Furthermore, limited information exists concerning BCS of aged pregnant mares following the onset of arginine supplementation and it is well known that, throughout the equine industry, the BCS scoring system (Henneke et al., 1983, 1984) is a subjective means to assess physiological state.

Intriguingly, it has been reported that dietary supplementation of arginine leads to a reduction in white adipose tissue in humans, pigs, and rats (Jobgen et al., 2009; Wu et al., 2009; Tan et al., 2012; Boon et al., 2019). Arginine enhances lipolysis in adipocytes, effectively mobilizing adipose tissue reserves (Wu et al., 2007; Jobgen et al., 2023). In obese rats, dietary supplementation with arginine beneficially stimulates the expression of genes for mitochondrial biogenesis and, thus, the oxidation of fatty acids in white adipose tissue (Fu et al., 2005). Similarly, partly through activating the adenosine monophosphate (AMP)-dependent protein kinase and the production of carbon monoxide, arginine supplementation promotes the oxidation of both fatty acids and glucose in rat skeletal muscle and liver (McKnight et al., 2010; Jobgen et al., 2022a, b).

In the current study, RF thickness and percent body fat mirrored changes in BCS resulting in a diet × day interaction for both RF thickness and percent body fat as both decreased in response to dietary arginine supplementation throughout the entirety of gestation. Although not analyzed in the current study, arginine supplementation has been shown to increase insulin production by beta cells of the pancreas of ponies (Holdstock et al., 2004). Furthermore, insulin secretion is reduced following the long-term supplementation of arginine in male Hannover Wistar rats (Mullooly et al., 2014), possibly due to amino acid imbalances or antagonisms (Wu, 2022). Robles et al. (2018) reported that 100 g/d of arginine increased insulin sensitivity in primiparous pregnant mares when compared to nonsupplemented mares. Although RF thickness did not differ due to dietary treatment at parturition, the increased sensitivity to insulin could explain the gradual decrease in RF thickness for ARG mares during mid-gestation. Maternal insulin resistance is a physiological adaptation to pregnancy that is needed to enhance the maternal supply of glucose to the fetus (Freinkel et al., 1974), disrupting alimentary glucose from being stored as fat by the mare. However, at parturition, there were no differences in RF thickness in response to dietary treatment. As arginine supplementation progressed through gestation in a “long-term” manner, we speculate that metabolic changes (e.g., lipolysis) occurred in the arginine supplemented mares to provide fatty acids as metabolic fuels. Further studies are warranted to test this hypothesis.

Color Doppler ultrasonography indices (RI and PI) are becoming a commonly used diagnostic tool to evaluate and quantify blood flow velocity to reproductive tissues via the uterine arteries in livestock species (Ginther, 2007). In the current study, there was no dietary influence on RI or PI throughout the initial 154 d of pregnancy. Although there was no effect of arginine on GUA blood flow during the initial 154 d, ARG mares experienced an increase in blood perfusion based on a decrease in RI in the GUA at parturition, perhaps due to an increase in synthesis of NO by endothelial cells in arginine-supplemented than in nonsupplemented mares. This decrease was expected as resistance to blood is known to decrease normally in gestating mares (Klewitz et al., 2015). Similarly, Mortensen et al. (2011) noted that arginine-supplemented mares (100 g/d) had greater blood flow (lower RI in the GUA) than control mares possibly due to maximal blood flow through the GUA. Because of the limited number of mares in the present study, a significant effect of dietary arginine supplementation on GUA blood flow was not detected using the Doppler ultrasound technique during the initial 154 d.

The supplementation of arginine to gilts between days 14 and 25 of gestation or between days 30 and 114 of gestation increases litter size and litter weight (Mateo et al., 2007; Li et al., 2014; Herring et al., 2022). Additionally, Chavatte-Palmer et al. (2018) reported that primiparous mares supplemented with 100 g/d of l-arginine during late gestation produced foals with heavier birthweights than foals from mares not supplemented with arginine. However in the present study, there was no effect of diet on BW, wither height, hip height, body length, or time to standing of foals prior to suckling or at 12 h postpartum. However, Mortensen et al. (2011) reported that arginine supplemented mares (100 g/d) had a significantly shorter gestation length when compared with nonsupplemented control mares. There was no effect of diet on gestation length in the present study, nor was there an effect of diet on placental weight, placental volume, or length of the umbilical cord. Robles et al. (2018) also found no effect of dietary arginine supplementation on weight, surface, or volume of the placenta, or wither height of foals at birth. The absence of differences in foal size in both studies may be attributed to a small sample size (*n* = 8, Robles et al., 2018; *n* = 5 present study). Additionally, there was no difference in time to expulsion of the placentae and all mares expelled the entirety of the fetal membranes within expected time period postpartum (Samuel et al., 1976; Ginther, 1992). Also, dietary supplementation of arginine did not affect the Brix % reading used to evaluate presuckle colostrum quality.

In gestating gilts and sows supplemented with dietary arginine, expression of VEGF, a signaling protein that enhances vascularization, was greater in their placentae compared to placentae of nonsupplemented sows (*n* = 9; Wu et al., 2012) (*n* = 10; Herring et al., 2022). In a similar study, larger fetuses from arginine-supplemented sows expressed more VEGF than intrauterine growth restricted fetuses (*n* = 9; Liu et al., 2012). However, a dietary difference was not observed on positive staining for VEGF, following histological analysis of the placental horn in the current study likely attributed to the small sample size (*n* = 5). Interestingly, Robles et al. (2018) observed the gene expression of SLC7A1 increased in placentas from primiparous mares that were not supplemented with arginine. Of which was similar to the current study, as arginine supplementation did not have an impact on positive staining for SLC7A1 following histological analysis of the placental horn. Again, the small sample size of the present study (*n* = 5) likely attributes for the absence of dietary differences.

In conclusion, results of the present study indicate that dietary arginine supplementation (50 mg/kg BW/d) is safe for gestating mares and may promote mobilization of white adipose tissue as indicated by lower BCS, RF thickness, and percent body fat throughout the entirety of gestation. Furthermore, the statistical differences observed for BCS, RF, and percent body fat did not negatively affect the health or size of the foals in this study. Future studies are needed with a greater number of mares to fully assess effects of supplemental dietary arginine on embryonic/fetal survival and growth in mares, specifically differences in blood flow and nutrient transport. However, results of this study suggest that maternal nutrition affects the cellular and molecular mechanisms of placental function, such as changes in resistance blood flow via the GUA prior to parturition.
